# Nutritional and non-nutritional dietary factors affecting obesity and chronic related disorders

**DOI:** 10.1093/eurpub/ckac129.356

**Published:** 2022-10-25

**Authors:** G Grosso, M Bonaccio

**Affiliations:** 1 Department of Biomedical and Biotechnological Sciences, University of Catania, Catania, Italy; 2 Department of Epidemiology and Prevention, IRCCS Neuromed, Pozzilli, Italy

## Abstract

There is convincing evidence that diet quality represents one of the most important public health targets to prevent non-communicable diseases. Dietary risks alone have been estimated to be responsible for about 10 million deaths due to cardiovascular diseases, metabolic disorders, and certain cancers worldwide, with trends not expected to decrease. Results from meta-reviews and expert assessments are quite in line with robust key messages to deliver to the general public. From a nutritional point of view, lack of fibre, vitamins and antioxidants, and unsaturated fatty acids together with high content in free added sugars, sodium, and saturated/trans fats represent key features for unhealthy dietary patterns and are responsible for most diet-related non-communicable diseases. While the role of animal-derived foods is still controversial due to the registered excess consumption and the environmental impact, a general low intake of plant-derived foods remains a major detrimental factor for health. The concept of diet quality should thus overcome the over simplistic calorie-count when coming to prevent obesity and related non-communicable diseases, while an adequate communication on this matter to the public is highly warrant. Together with these general dietary recommendations relying on nutritional aspects affecting human health, emerging evidence is now also exploring the role of non-nutritional dietary factors. The level of processing, the alteration of the food matrix, the presence of additives (such as flavor enhancers, colorants, emulsifiers, artificial sweeteners, thickeners, foaming/anti-foaming agents, and preservatives) have been demonstrated to play a role in metabolic health and inflammatory-related disorders. These aspects are currently under investigation, but they might represent an underrated concern for public health due to the food market drive and a perfect fit into the obesogenic modern society.

